# Hydrogel, Electrospun and Composite Materials for Bone/Cartilage and Neural Tissue Engineering

**DOI:** 10.3390/ma14226899

**Published:** 2021-11-15

**Authors:** Beata Niemczyk-Soczynska, Angelika Zaszczyńska, Konrad Zabielski, Pawel Sajkiewicz

**Affiliations:** 1 Institute of Fundamental Technological Research, Lab. Polymers & Biomaterials, Polish Academy of Sciences, Pawinskiego 5b St., 02-106 Warsaw, Poland; bniem@ippt.pan.pl (B.N.-S.); azasz@ippt.pan.pl (A.Z.); 2 Faculty of Materials Science and Engineering, Warsaw University of Technology, Woloska 141, 02-507 Warsaw, Poland; konrad.zabielski1001@gmail.com

**Keywords:** scaffolds, tissue engineering, polymers, electrospun nanofibers, hydrogels, nanoparticles, composites, injectable materials

## Abstract

Injuries of the bone/cartilage and central nervous system are still a serious socio-economic problem. They are an effect of diversified, difficult-to-access tissue structures as well as complex regeneration mechanisms. Currently, commercially available materials partially solve this problem, but they do not fulfill all of the bone/cartilage and neural tissue engineering requirements such as mechanical properties, biochemical cues or adequate biodegradation. There are still many things to do to provide complete restoration of injured tissues. Recent reports in bone/cartilage and neural tissue engineering give high hopes in designing scaffolds for complete tissue regeneration. This review thoroughly discusses the advantages and disadvantages of currently available commercial scaffolds and sheds new light on the designing of novel polymeric scaffolds composed of hydrogels, electrospun nanofibers, or hydrogels loaded with nano-additives.

## 1. Introduction

Tissue engineering (TE), as an interdisciplinary field of science, is focused on employing modern technologies to design and develop particular biomaterials, i.e., implants or scaffolds, to improve and maintain desired tissue and restore their functions. Biomaterials play a key role in TE applications since they can support cell proliferation, differentiation, attachment and neo-tissue genesis [1]. With the increasing knowledge concerning interactions between cells and the surrounding local environment, attention is predominately directed toward developing the biomaterials which can mimic the extracellular matrix (ECM) components, including its properties and complex structure [2]. So far, an important number of biomaterials, including hydrogels and electrospun porous scaffolds, have been widely investigated and tested in tissue engineering applications [3].

**Figure 1:**
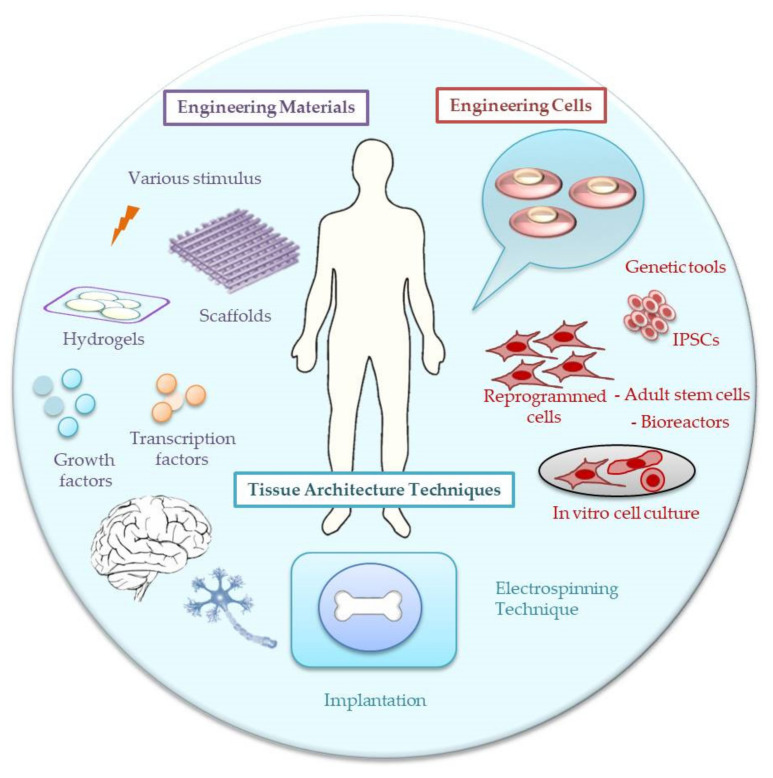
Engineering material, cell and tissue architecture techniques dedicated to the tissue engineering applications.

Many publications report the general scaffold requirements defined by tissue engineering [4,5,6,7]. The most prominent requirements include biocompatibility, non-toxicity, controlled biodegradation rate, adequate porosity and swelling behavior stability in the physiological environment, ability to vascularization, appropriate morphology that provides cell infiltration, adequate biochemical and mechanical properties providing appropriate cell–material interaction (Figure 1).

More specific demands are rather individual depending on the dedicated tissues. Bone/cartilage and neural tissue engineering are still highly demanding in regards to biomaterials. Besides the above-mentioned prominent requirements, biomaterials dedicated to bone/cartilage should resist various stresses, e.g., compressive stress, corresponding to the magnitude characteristic of various cartilage areas, which are discussed in more detail below. However, during biomaterial designing it must be considered that stress values depend on sex, age, the individualistic nature of a gait, and biochemistry [8]. Both bone/cartilage and neural tissue engineering require the plasticity of the material that will precisely fill the cavity and be introduced into the body in a convenient, minimally invasive way—for instance, by injection. Neural tissue engineering imposes another requirement on biomaterials; they should be electrically conductive to mimic neurotransmission, thus providing adequate communication between neuronal cells adhering to the material [9].

Over half a million bone graft treatments are conducted yearly in the US, making it the most transplanted tissue after blood [10]. Osteoporosis is one of the leading disease-related causes of bone fracture for people above the age of 50. Based on a study conducted in six European countries, fragility fractures are expected to increase by 23.3% between 2017 and 2030, with annual costs increasing by 27% [11]. Osteoarthritis affects 10.5% of the US population, and in 2019 their treatment costs exceeded $300 billion [12]. In 2017 over 300 million people globally were diagnosed with osteoarthritis [13]. It is estimated that over the next 20 years, this number will increase by 50% [14].

Regarding neurodegenerative diseases, they have a considerable impact on the social and economic aspects of many people’s lives. It is stated that global medical and social care costs caused by dementia in 2019 reached US $1.3 trillion [15]. It is estimated that the overall number of people affected by dementia will be 75 million by 2030 and over 130 million by 2050 [16]. Currently approved treatments focus on symptom suppression but often show no beneficial effect on patients’ quality of life [17]. Moreover, traumatic spinal cord injury and traumatic brain injury affect 13 and 369 people per 100,000 population every year, respectively [18]. 

Diseases and injuries of bone/cartilage tissues and the central nervous system still cause global socio-economic problems. It is clear that therapies providing tissue regeneration are a real need, but designing biomaterials dedicated to those tissues is a tremendous challenge. Thus, this comprehensive review thoroughly discusses the state-of-the-art of currently available materials as well as technological innovations of scaffolds for this complicated tissue regeneration. Particular attention will be paid to injectable hydrogels loaded with nano-additives, i.e., nanoparticles or electrospun fibrous scaffolds. In this regard, the advantages, disadvantages and future perspectives of such materials dedicated to various fields of tissue engineering are thoroughly discussed.

## 2. Requirements for Scaffolds for Tissue Engineering Applications

Biocompatibility is one of the most crucial properties; it refers to the ability of a biomaterial to perform its desired function without eliciting any undesirable local or systemic response [19]. The human immune system can be divided into two subtypes: The innate immune system, which includes polymorphonuclear cells, mononuclear phagocyte cells and lymphocytes and is responsible for detection of intruding agents and triggering immune responses, whereas the adaptive immune system consists of lymphocytes (B and T cells), is activated by the innate immune system and is responsible for the elimination of pathogens and immunological memory of the host [20,21]. However, while removal through phagocytosis is unsuccessful, the mammalian organism answers with a foreign body response (FBR), which according to the current knowledge happens in five phases: Protein adsorption, acute inflammation, chronic inflammation, foreign body giant cell formation and fibrous capsule formation [22]. Nonetheless, many immunomodulation methods have been used to improve inflammatory response by modifying physical properties, surface chemistry, controlled release of active compounds and cell therapy [23,24,25], some of which focus on deactivation or induction of M1 or M2 macrophages, respectively.

Another property imposed by TE on biomaterials is biodegradation ability. Polymeric materials can be classified based on their origin as synthetic or natural. In recent years natural biomaterials have gained a lot of interest. They have unique properties such as excellent biocompatibility, non-toxicity and adequate biodegradability [26]. However, their complex molecular structure limits control over physicochemical properties. The isolation of these materials from animals or plants provides difficulties in their purification and total removal of pathogens and viruses [27]. Many natural polymers rapidly dissolve in water, which limits their scope of applications in TE. To overcome this problem, many of them are treated with crosslinking agents to provide adequate mechanical properties, stability in physiological conditions and controlled degradation rate [28]. Nevertheless, such commonly used crosslinkers as glutaraldehyde or formaldehyde are toxic in contact with cells, whereas their complete removal after the process is often impossible [29]. Currently investigated alternatives are physical crosslinking methods, which induce physical bonding under specific environmental changes (e.g., temperature, pH, UV) [30]. While these methods have been proven to be viable for many materials used as scaffolds for drug delivery systems, materials crosslinked this way have poor mechanical properties, as an effect of non-covalent, relatively weak bonding between polymeric chains. Photo- and enzymatic-crosslinking methods provide better strength than physical crosslinking. However, there are still concerns regarding cytotoxicity of free radicals produced with photoinitiators [31] and enzymes themselves [32]. On the other hand, synthetic polymers are often insoluble in water and, for some processing methods, chemical solvents are required. Similar to crosslinkers, their residual presence is undesired due to their cytotoxicity. It was shown that polar solvents are less cytotoxic to L929 cells compared to nonpolar ones [33]. While many electrospinning processes reported in the literature are solvent based, there are available alternatives like melt, supercritical CO_2_-assisted, anion-curing, UV-curing, thermocuring electrospinning methods that do not need solvent [34].

Providing a 3D porous structure is crucial for nutrient and gas transportation and effective cell infiltration inside the scaffold. Adenosine-5’-triphosphate (ATP) is the natural energy storage of the cell, and the lack of it leads to cell necrosis. In hypoxic conditions, cells produce ATP through lactic acid fermentation, which uses 15 times more glucose than oxidative phosphorylation [35]. However, stem cells seem to be more resilient to hypoxia than their progenies. MSCs in mild hypoxia show lower necrosis and cell injury, higher proliferation and preferential differentiation into osteocytes instead of adipocytes, but their metabolism is slowed down, and migration capability is affected [36]. A hypoxic microenvironment influences specific stem cells in different ways, and studies show ambiguous results but the majority agree that hypoxia plays a key role in maintaining stemness and affects differentiation phenotype [37,38]. 

Mechanical properties that correspond to dedicated tissue might improve cell adhesion and, at the same time, precisely fill the injured tissue. It is known that matrix stiffness influences the differentiation of stem cells. Adequate biochemical properties occur by specific protein sequences presented in native ECM and increase effective interactions between scaffold and cells. In this respect, fibrous proteins like collagens, fibronectin, elastin and fibrillin are the proteins that are common in native ECM. Their primary purpose is to provide tensile strength and elasticity for the ECM [39]. Various proteoglycans present in native ECM bind water molecules and create hydrated structures able to resist compressive forces. Moreover, surface charge showed to modulate protein adsorption and cell adhesion [40], and the type of charge a protein is attracted to depends on the protein’s isoelectric point [41]. 

However, each tissue fulfills different functions and hence characterizes different morphology, biochemistry, stiffness and physiology. Taking this into account, the actual requirements placed on scaffolding are individual to the specific type of tissue.

### 2.1. Bone Tissue Engineering

Scaffolds dedicated to bone tissue engineering should be biocompatible, degrade with the rate matching the formation of new tissue and possess porous, three-dimensional structure [42]. Moreover, scaffolds should promote osseointegration, be osteoinductive, provide osteogenic factors and anti-infectives and carry cells [43]. For tissue ingrowth, an interconnected open porous structure with macropores (>100 μm) and micropores (<20 μm) is required as optimal [44]. However, it is recommended that macropore sizes should be greater than 300 μm to provide a larger surface area and promote cell infiltration [45]. The bone extracellular matrix is a very complex and complicated structure due to its architectural and functional diversity [46]. The functional bone should include the fusion of the native bone functions and healed bone with the addition of the host bone. This kind of structure is significant for the high strength needed and load-bearing applications. In both cases, mesenchymal cellular condensation occurs firstly that acts mainly as a platform for osteogenesis. Cells can differentiate in osteoblasts. Moreover, endochondral bone formation can form a substantial portion of bone. This method includes differentiation of mesenchymal cells into the chondrocytes reliable for the deposition of cartilage regeneration and can be later mineralized and substituted with bone [47,48,49]. However, the method has been tested in the specified period without testing the long-term results of the bone healing process. Bone tissue is a specific type of self-healing tissue after most of the fractures. However, fractures with a more complex nature or some diseases can limit this capacity [50]. Rachit et al. reported that after injury of the bones’ stromal site, near to the cartilage area, chondrocytes mortality increases and those that managed to survive showed a severe metabolic dysfunction. The healing process is additionally limited by the gender and age of the patient. Introduction of natural materials, such as silk, chitosan, agarose and many others [51], could be an interesting solution. More investigation is needed to understand this process. 

**Figure 2:**
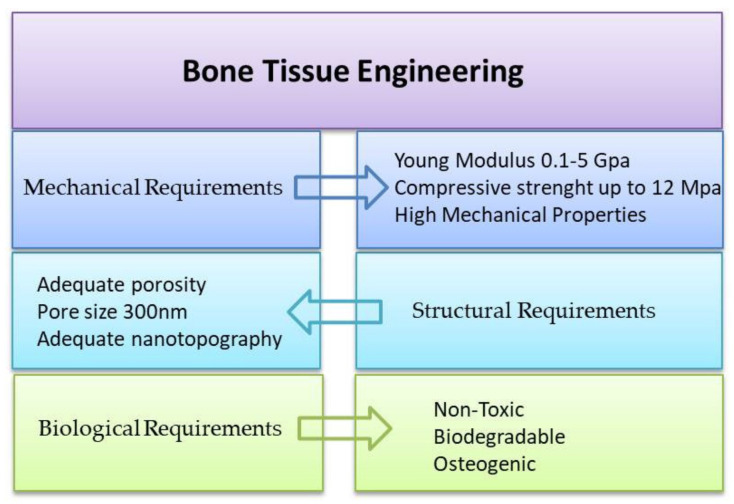
Scaffold requirements for the bone tissue engineering applications.

Mechanical properties of the scaffold should correspond to the target tissue to diminish the chance of negative outcomes like stress shielding, osteopenia and bone refracture [52]. Osteoblasts on the scaffold with stiffness lower than 100 kPa (non-mineralized bone) show no evidence of mineralization. Still, while seeded on stiffer substrates, the amount of mineral deposits significantly increased with increasing Young’s modulus. The maximal differentiation of osteoblasts was reached when the scaffold modulus corresponded to the mineralized bone tissue, which was c.a. 300 kPa or higher [53]. According to Poumarat et al. [54] the stiffness of the scaffold dedicated to the bone, tendon and ligaments tissue engineering should be higher than the biological structures occurring in our body, even if it comes at the cost of less biocompatibility. However, then it might cause an undefined inflammatory response and, in effect, implant rejection. Scientists are still working on scaffolds that combine good biocompatibility and strong mechanical properties resulting in the successful replacement of damaged tissue. One of the methods is incorporating growth factors like BMP-2, BMP-7, TGF-β2, etc., into the scaffolds, showing adequate mechanical properties. Such factors are responsible for the stimulation of wound healing and tissue repair; therefore, they should be easily accessible for the cells on the scaffold’s surface and not placed inside the polymer core [55]. The main scaffold requirements dedicated to the bone tissue engineering are presented in Figure 2.

Besides the requirements above, scaffold dedicated bone/cartilage tissue engineering should be focused on controlled biodegradation rate, adequate flexibility allowing fill and match to the cavity, and minimally invasive way of introduction to the body.

### 2.2. Neural Tissue Engineering

Compared to all tissues discussed in this review, neural cells have the poorest regenerative ability. While peripheral nerves are able to regenerate injured axons in a short range, the regeneration of central nervous systems (CNSs) is not that easy to recover [56]. This is due to the complex structure, fulfilled advanced functions of cells and the occurrence of reactive astrogliosis. Seconds after an injury of CNS, a cascade of events occurs, leading to inflammation; over a span of 2 to 10 days, astroglial scar tissue forms, and the acute stage ends with tissue remodeling in the vicinity of the lesion [57]. These cellular and architectural changes create a microenvironment, which prevents axons from regenerating [58]. Properties of cell-laden scaffolds must be properly tuned to provide support in nervous tissue regeneration. The scaffold’s stiffness affects cell adhesion, growth and differentiation, and optimally should match the target tissue’s. For example, multipotent mesenchymal stem cells (MSCs) differentiate into neurogenic, myogenic or osteogenic lineages based on the affinity of the substrate’s elasticity to brain, muscle and bone tissue’s elastic modulus, respectively [59]. Many studies point out that cells prefer microenvironments that have closely related biomechanical properties to their native tissue. Neural stem cells (NSCs) on extremely soft substrates (~10 Pa) do not adhere to the matrix and remain rounded, and clustered [60]. NSCs and human neural progenitor cells (hNPCs) both show preferential differentiation into elongated neuronal cells in the lower spectrum of the brain’s elasticity (Ebrain ~ 0.1–1 kPa) and into star-shaped glial cells on more rigid matrices [59,61]. Different surface topographies at micro and nanoscale prove to guide orientation and growth of neuronal and neuroglial cells along the axis of continuous (grooves, fibers) and discontinuous (pillars, posts, cones) structures [62]. The incorporation of peptide motifs provides places for focal adhesions. The most commonly investigated integrin-binding peptide sequences are fibronectin-derived tripeptide Arg-Gly-Asp (RGD) and laminin-derived Tyr-Ile-Gly-Ser-Arg (YIGSR) and Ile-Lys-Val-Ala-Val (IKVAV) [63]. Moreover, YIGSR and IKVAV sequences showed to support neurite outgrowth [64,65]. Different peptides are also tested; for instance, neuroprotective short peptide (NAPVSIPQK) provided neuroprotection and promoted neurite outgrowth, surprisingly without any growth factors [66]. Modification of scaffolds with different chemical functional groups affects differentiation phenotype and migration. NSCs cultured on surfaces with -NH2 and -COOH groups exhibit preferential differentiation into neurons and promote migration, whereas on -OH surfaces, cells barely migrated and differentiated only into glial cells [67]. Naturally, electrical signals play a crucial role in neuron functioning. It was proven that electrical stimulation affects NSPC migration [68] and promotes peripheral nerve regeneration [69], thus conductive (and piezoelectric) materials gained a lot of interest for nerve tissue engineering applications [70,71]. Through incorporation into scaffold growth factors (i.e., insulin-like growth factors (IGF), vascular endothelial growth factor (VEGF) and neurotrophic factors like nerve growth factor (NGF), brain-derived neurotrophic factor (BDNF), neurotrophin-3 (NT-3), neurotrophin-4/5 (NT-4/5)), the proliferation, differentiation and guidance of neuronal and glial cells can be improved [72,73]. Lastly, due to the risks involved in CNS tissue treatment, scaffolds should be designed so that the procedure is minimally invasive; thus injectable hydrogels seem like the best option.

**Figure 3:**
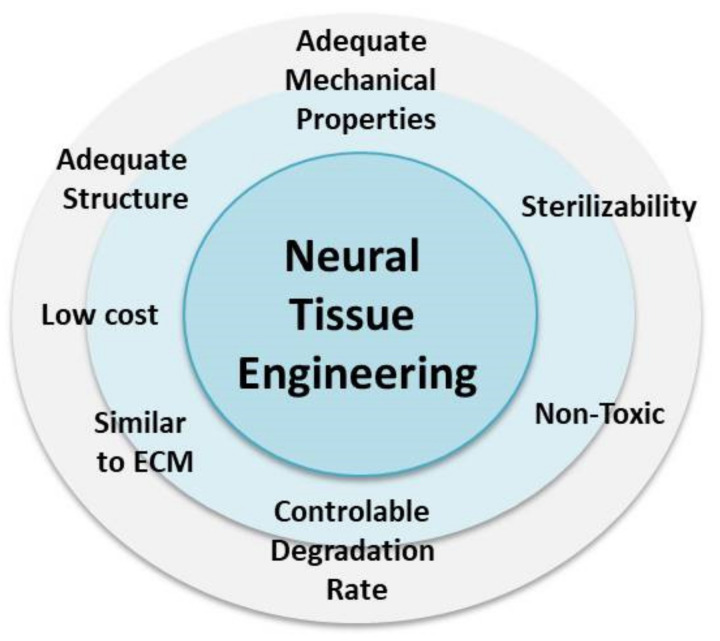
Scaffold requirements for the neural tissue engineering applications.

Electrical stimulation provided by the scaffold is another essential requirement that a scaffold should fulfill. It is especially important from the point of view of nervous system signal transductions between neurons [74]. In the human brain, electrical conductivity is in the range of c.a 0.6–2.4 mS/cm, occurring with the signal transduction of c.a. 0.7 km/s [75]. By using electrically conductive scaffolds mimicking native neurotransmitters, not only could neurons regenerate, but original neuronal functions could also be restored. Electrical stimulation is still challenging due to the complexity of the neuronal transmission process, providing an adequate time for such stimulation. Additionally, currently available materials showing desired electrical properties usually are non-degradable, non-biocompatible, non-compliant with neural tissues in terms of structure, biochemical or mechanical properties, and usually show inflammatory character [76,77]. The main scaffold requirements dedicated to the neural tissue engineering are presented in Figure 3.

## 3. Hydrogels Dedicated to the Tissue Engineering Applications

**Figure 4:**
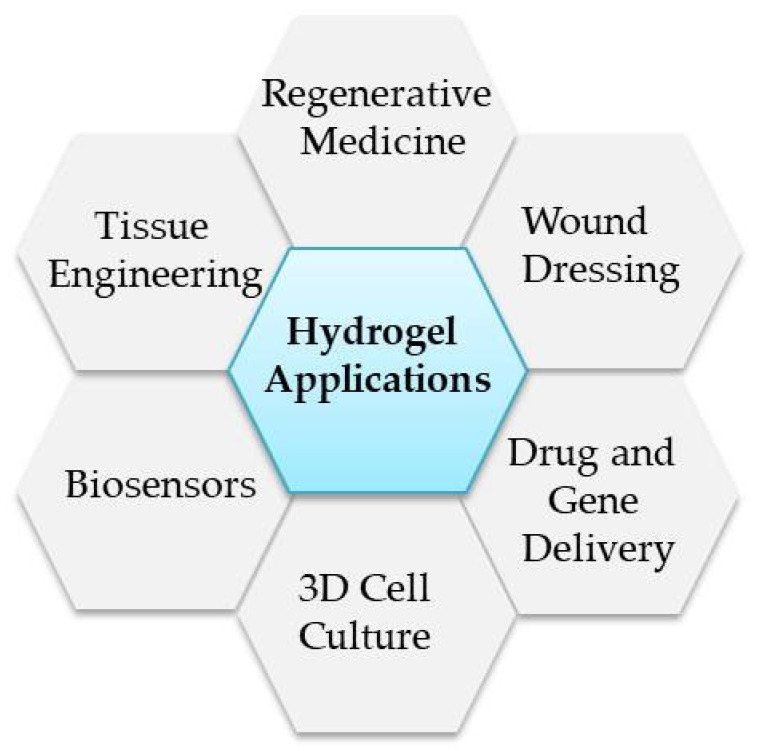
The main hydrogel applications.

Hydrogels belong to very promising materials that might serve as scaffolds used for cell culture studies. They are polymers that form 3D highly hydrated polymeric networks [63,78]. Hydrogels are very attractive from the perspective of tissue engineering due to their biocompatibility, easy processing ability and structural resemblance to native ECM [63]. Various methods of hydrogel classifications might be distinguished. They might be classified in terms of origin to natural, synthetic and hybrid hydrogels. The cellulose and its derivatives, carrageenan, sodium alginate, agarose, chitosan, dextran, hyaluronic acid (HA), fibrin, collagen and gelatin [79,80], are just a few important examples of natural representatives of hydrogels, while poly(methyl methacrylate) (PMMA), polyethylene glycol (PEG) and poly(2-hydroxyethyl methacrylate) (poly(HEMA))-based polymers [81] belong to the synthetic sort. The examples of hybrid hydrogels are gelatin-PEG [82] and chitosan/acrylamide [83]. Hydrogels also might be divided in terms of ionic charge to cationic (e.g., chitosan) [84], anionic (e.g., HA) [85], amphiphilic (e.g., collagen) non-ionic hydrogels (e.g., dextran) [86]. Another division assumes hydrogels’ biodegradation ability. The polysaccharide-based or protein-based hydrogels are biodegradable, while acrylate-based hydrogels belong to non-biodegradable hydrogels [87,88]. One of the most common hydrogel classifications is crosslinking methods that might occur either chemically or physically. The main hydrogel applications are presented in Figure 4.

Hydrogels might also be divided considering their applications. In tissue engineering, they might be used as drug, cell or growth factor delivery systems, biosensors as well as scaffolds [63,80,85]. The last method of hydrogel classification includes their implementation into the body: They might be non-injectable or injectable. Currently, many commercial injectable hydrogels serve as scaffolds for tissue engineering, e.g., EUFLEXXA^®^, HyStem^®^ [89], Corning^®^ Matrigel^®^ [90], Biogelx™ [91] and others. The details are presented in Table 1.

There exists a unique group of hydrogels that show changes in swelling behavior, degradation rate, electrical conductivity or crosslinking under external stimulation. They are so-called stimuli-responsive (smart) hydrogels [92,93]. In this context, the hydrogels might be divided into conventional or stimuli-responsive ones—the response to external stimuli, which might occur physically, chemically or biochemically. A great example of such an approach is the synthetic commercially available Mebiol^®^ gel. It consists of poly(N-isopropyl acrylamide) and poly(ethylene glycol) (PNIPAAm-PEG), which belong to the group of the thermo-reversible gelation polymers (TGP) [94]. While, with heating, such an approach transforms sol to gel, this phenomenon reverses while temperature decreases. Such TGP hydrogels might serve as scaffolds for in vivo cell regeneration [95,96]. Another example of stimuli-responsive polymeric materials is shear-thinning hydrogels, such as hyaluronic acid (HA) [97]. Their unique rheological properties result from long polymeric chains that form random coils and gel due to molecular entanglements. Under sufficient shear force, the polymer molecules undergo disentanglement and alignment, leading to a large shear thinning effect when sufficient high molecular weight polymer is used. Such innovative properties allow the injection of high-viscous hydrogels [4]. Such properties of the biomaterial as thermal sensitivity or shear-thinning behaviour enjoy significant interest, especially as injectable scaffolds for bone/cartilage and neural tissue engineering. 

Since bone/cartilage tissue regeneration is a complex and multi-step process, the requirements imposed on the potential scaffold are relatively high. On the one hand, hydrogels are very attractive while considering cartilage regeneration, not only due to the three-dimensionality of their structure and physical resemblance to the cartilage ECM. Mainly, hydrogels, which might be introduced to the injured cartilage by injection, generate minimal after-surgery scar. This feature makes them beneficial from the perspective of economy, convalescence rate and diminished after-surgery complications. However, there are still some crucial requirements that must be met before putting them into clinical practice. One of the most important is to ensure cross-linking of sol after injection as quickly as possible to avoid material leaking out of the lesion as well as to provide sufficient mechanical stability of the scaffold. So far there are no commercial injectable scaffolds approved for cartilage regeneration and intensive research is underway to introduce such material to clinical practice. Some injectable hydrogels have recently undergone clinical trials [98]

An additional problem is related to the shear stresses accompanied by moving which may lead to the destruction of the hydrogel 3D structure through the shear thinning mechanism [99]. Additionally, clinically used hydrogels, especially synthetic ones, are biologically inert, resulting in reduced cell–scaffold interactions, unstable physiological conditions and poor mechanical properties [100]. These features currently make existing hydrogels (e.g., Pluronics) short-term solutions. Naturally derived hydrogels such as hyaluronans show improved bioactivity; they effectively bind with chondrocytes through CD44 and RHAMM receptors [4]. An interesting approach is the hydrogel system which combines thermosensitivity of methylcellulose (MC) with shear-thinning behavior of HA. Additionally, HA interacts effectively with chondrocytes through CD44 and RHAMM receptors that provide stem cell migration participating in cartilage formation, see [4]. It has also been reported that the presence of HA provides up to 80% of native cartilage ECM formation as well as chondrocytes retention and infiltration after two weeks in the rabbit osteochondral defect model [101]. 

Nevertheless, the high water solubility of hyaluronans leads to rapid dispersion after being injected into fluid-filled cavities. The partial crosslinking of hyaluronans solves this problem to some extent but does not overcome their poor mechanical properties regarding cartilage requirements or rapid biodegradation profile [102]. Commercially used hyaluronans only moisturize the damaged tissue and reduce the friction in articular cartilage for a while but do not provide adequate integrity with healthy areas of cartilage, and do not supply the nutrients and gas exchange [103]. Consequently, current strategies using hydrogels for bone and cartilage tissue engineering need the use of additives that significantly increase the mechanical properties of the hydrogel and biological factors that provide effective regeneration of bone/cartilage tissues. Most of the current promising approaches for such therapies involve hydrogels/nano-additive systems, which are described in the next section.

The hydrogels seem to be excellent candidates for CNS injuries, especially in situ thermosensitive gelling ones that can be injected [104]. The conventional scaffold implantation by complex surgeries leads to blood–brain barrier damage, resulting in fluid and blood cell infiltration into the ventricle and subsequent inflammation and immune response [105]. 

Hydrogel injection can be conducted efficiently to the spinal cord or brain cavities, providing in situ gelations and filling the irregular lesion. At the same time, such hydrogel might ensure adequate support for cell growth and deliver drugs, growth factors and cells directly to the needed place without violating the blood–brain barrier.

Additionally, hydrogels mechanically resemble soft tissues, such as the brain, more closely than stiff ones. Consequently, it is required to provide either mechanical properties supporting cell adhesion without cytotoxic effect and relatively fast in situ gelations. Usually, these properties do not correspond to each other. Thonhoff et al. [106] tested various commercially available scaffolds for human neural stem cell (hNSC) differentiation dedicated to an injured brain or spinal cord. In these studies, Pluronic, Martigel and Pura Matrix were tested. The WST-1 cytotoxicity differentiation capacity results showed the Pluronic at the concentrations providing adequate in situ gelation was simultaneously toxic to hNSCs. Among others, Pura Matrix showed the lowest cytotoxicity to hNSCs, satisfactory cell adhesion with sufficient gelation capacity and, most importantly, the best cell survival, migration and differentiation. These studies show the importance of keeping the balance between gelation rate and adequate hydrogel stiffness; these requirements will vary depending on the dedicated part of tissue but are undoubtedly important, especially from a practical point of view.

In other studies, a non-commercial hydrogel with interesting properties from the neural tissue engineering point of view was designed. Rinoldi et al. [107] synthesized a smart conductive semi-interpenetrating polymer network (semi-IPN) poly(N-isopropylacrylamide-co-N-isopropylmethacrylamide)(P(NIPAm-co-NIPMAm))/polythiophene-based hydrogel for regeneration of neural tissues. Compared to pure (P(NIPAm-co-NIPMAm) hydrogel, the composite material showed three-fold decreased impedance, corresponding to increased electrical properties. The Atomic Force Microscope (AFM) nanoindentation analysis showed Young’s modulus of composite fell within the required range of neural tissue and was c.a. 5 kPa. In vitro studies on mesenchymal stem cells and neural progenitor cells also confirmed the great potential of such an approach. The fluorescent microscopy showed that neural progenitors more efficiently differentiated into neural cells, with higher amounts seeded on composite scaffolds than pure hydrogel.

To sum up, despite their many advantages, hydrogels might show various stability and gelation times in vitro and in vivo [108]. Additionally, most hydrogels, especially synthetic ones, characterize biological inertia due to a lack of sites that effectively bind cells with material, resulting in decreased adhesion and proliferation [109]. The hydrogels are also unstable in physiological conditions, resulting in faster than expected degradation in vivo. 

Considering the requirements of bone/tissue engineering, hydrogels occurring alone cannot withstand mechanical stresses existing, i.e., in articular cartilage. These features currently make clinically available hydrogel solutions a temporary treatment that only mimics synovial fluid in the cartilage tissue, which decreases the friction in articular cartilage for a very short time. 

Based on CNS tissue engineering requirements, natural hydrogels hardly provide electrical conductivity by themselves [110]. This problem might be overcome by the incorporation of nano-additives that provide electrical cues. Another method is designing and synthesizing synthetic hydrogels, e.g., smart synthetic semi-IPN hydrogels [107].

## 4. Electrospun Scaffolds Dedicated to Tissue Engineering

Electrospun nanofibers are formed using an inexpensive and simple method that allows to process a wide spectrum of natural or synthetic polymers [120]. The properties of electrospun mats might be easily controlled by choosing adequate material and processing parameters, i.e., flow rate, electric field, the distance between needle and collector, etc. Electrospun nanofibers fit some of the requirements of TE application—due to fibrous, architecture they are able to withstand the stresses occurring in various tissues, i.e., compressive or tensile stresses in cartilage [121]. A high surface to volume ratio of electrospun nanofibers and high porosity are other features that make them attractive for TE. Such architecture faithfully reflects porous collagen fibers that are present in native ECM. Additionally, electrospun nanofibrous scaffolds might be formed using a wide range of biocompatible polymers, making this technique very attractive [122]. There are various types of commercially available electrospun scaffolds with specific directions of applications. The most commercially used scaffolds are, e.g., TissueMend R (USA), PolyTape R (Japan), OrthAdapt (USA) and many others [122,123].

While designing adequate mechanical properties of the scaffolds, the material selection has to be done thoroughly. Since all medical implants, such as PGA and PLA, need to be sterilized, e.g., via radiation, gas or steam, it can affect and decrease the mechanical properties of the material [124]. Thus, the sterilization method should be selected individually with respect to selected material. Moreover, implanted scaffolds are exposed to various mechanical loadings, such as shear tension, torsion and compression. The mechanical strength of cortical bone is around 100–230 N [125], tendons and ligaments 1978 ± 301 N [122]. Mechanical tests characterized that the load to failure is 76 N, 38 N and 229 N for TissueMend, Restore and GraftJacket, respectively [126]. In this respect the scaffolds with similar mechanical strength, that withstand the natively occurring mechanical stages, to the GraftJacket should be formed. Another method to reinforce mechanical properties of the synthetic scaffold, such as PLA, PGA, and their copolymers, is formation of composite scaffolds by incorporation of other materials, i.e., ceramic materials, bioactive glass or hydroxyapatite (HAp) into the polymer matrix.

The controlled scaffold degradation after implementation also plays an important role. The ideal scaffolds after tendon or bone regeneration should ultimately degrade in 12 months, because this is approximately the longest time of these structures’ regeneration. Based on the available literature, the degradation process differs dramatically. Synthetic scaffolds such as commercially available anterior cruciate ligament (ACL) degrade slowly or not at all [127]. Restore Patch was degraded in in vivo studies after 112 days after implementation. TissueMend and GraftJacket degraded partially, while Zimmer Patch was non-degradable. BioBlanket scaffold is expected to degrade in in vivo conditions for up to one year [128]. Debnah et al. showed that synthetic scaffolds are current in host tissue even after 15 years [129]. The tissue induction is not well defined: Zimmer Patch was surrounded by tissue capsule; CuffPatch was partially replaced by host tissue. Acid products formed during the degradation process can affect the reduced proliferation of the host tissue. Researchers, such as Guidoin et al., [127] examined the ACL prosthesis healing process and found that healing is poor, incomplete and unpredictable. The complex system used today is the Ligament Advanced Reinforcement System (LARS) made from polyethylene polyester fibers. Fibers are twisted at 90° and it gives higher mechanical properties. In vitro tests on human fibroblasts showed cellular growth after six months culturing on LARS [130]. Clinical tests concluded that LARS can be used for ACL reconstruction [131]. 

Most clinical studies are case and retrospective studies. Additional research studies are necessary to precisely prove the safety and efficiency of commercial scaffolds in respect to long-term use. In existing studies, selected criteria are often limited by age, gender and defect sizes. Selected examples of commercially used scaffolds are presented in Table 2.

Using electrospun polymeric materials is more common in peripheral nervous system regeneration than for central nervous system therapies. Numerous collagen type-I scaffolds, dedicated neural tissue engineering, have FDA (Food and Drug Administration) approval, such as NeuroMatrix™, Neuroflex™ [148], Neuragen^®^ [149], Neurolac™ [150], NeuroTube^®^ [151,152], Salutunnel [153] and others [154,155,156,157]. They usually serve as nerve conduits and are formed into tubular structures to effectively bridge nerve gaps, preserve the injured nerve against scarring and guide the regeneration of axons by mechanical stimulation. In these materials, such parameters as scaffold stiffness, permeability and controlled degradation were well designed, and provided satisfactory efficiency without compression neuropathy [150]. However, the main challenge that meets commercially available materials dedicated to neural applications is the controlled degradation rate. When the degradation rates are incorrect, cell proliferation and migration are decreased, which causes a hindering regeneration of the scaffolds. Particular studies have shown that providing different amounts of collagen might affect different properties of the scaffolds, including degradation rate [158]. An example of commercial collagen scaffolds that show the controlled biodegradation is Neuragen. It dissolves after c.a. four years after implantation. Another method that might improve degradation rate is blending collagen with such synthetic polymers as PLA and PGA [159]. While improving the degradation rate, according to some of the scientific reports, i.e., [150], many of the commercial approaches are not able to provide full restoration of motor and sensory nerves in the place of injury, do not supply enough nerve fibers to stimulate the target organ and do not minimize muscle atrophy before nerve fibers grow into the organ [160]. This might be caused by the bioelectrical inertia of implanted materials. One of the solutions that might solve this problem is providing electrical stimulation by the scaffold. In this respect, the materials must be selected thoroughly to provide adequate degradation, mechanical properties and bioactivity and to avoid chronic inflammation, which are usually the results of using such electroconductive materials as Polyaniline (PANI). An interesting, innovative electroactive electrospun nanofiber that, by providing the adequate intensity of electrical stimulation, promotes nerve regeneration was reported by Zhang et al. [161]. A great example of such an approach is Antheraea pernyi silk fibroin (ApF)/(Poly(L-lactic acid-co-caprolactone)) (PLCL) electrospun nanofibers coated with reduced graphene oxide (RGO). Such modification increased mechanical properties and bioactivity, but most importantly provided long-term electrical conductivity comparable to that occurring in the human nervous system [75], which was c.a. 0.41 mS/cm. Moreover, such modification provided the steady interaction between neural cells/tissues and conductive scaffolds. The PC12 cells seeded on the AP/RGO nanofibers showed increased differentiation after RGO modification, while its in vivo implantation into rat sciatic nerve defects showed a comparable capacity regeneration to autograft, which is the current gold standard in peripheral nerve regeneration [162]. Such graphene-modified electroconductive electrospun nanofibers are a promising approach for peripheral nerve repair and regeneration.

Electrospun nanofibers present plenty of favorable advantages from the bone/cartilage or peripheral nerve tissue engineering point of view. Nevertheless, they offer a rather two-dimensional structure, while their implantation into injured tissue might be carried out by surgery only. This is a serious problem, especially while the injured site is challenging to reach or the surrounding area is sensitive to damage. To overcome this problem, an appropriate modification method might be used e.g., electrospun nanofiber cutting. Such modification might be conducted during the electrospinning process, or as a post-electrospinning treatment [163]. The former assumes adjustment of electrospinning parameters [164,165], use of electric spark [166] or core-shell electrospinning with subsequent leaching of the shell [167]. The latter might occur chemically (i.e., by aminolysis, hydrolysis), where polymeric chains are cleaved using diamines, acids or alkalisor physically by mechanical, laser or ultrasonic cutting [168]. The length of the nanofibers obtained this way might be in the range of 1–200 μm and by fiber dispersion in the adequate medium and then thorough adjustment of needle size, nanofibers in such a way might be easily injected into the lesion. The fiber modifications that make them injectable are the progressive steps forward designing new biomaterials and increasing the scope of applications of electrospun nanofibers. The details regarding such approaches are discussed below.

## 5. Composite Injectable Scaffolds

### 5.1. Hydrogels and Nanoadditives

Some hydrogel limitations could be overcome by appropriate modification. One of them is adding some nano-additives, e.g., nanohydroxyapatite, nano-silicate, polymeric micelles, liposomes and others [169,170]. In such systems, hydrogels provide a biocompatibility, biodegradation ability, three-dimensional structure with interconnected pores that fairly mimic native ECM or such smart properties as thermal sensitivity. Additionally they are the perfect medium for nano-additive dispersion. Nano-additives fulfill a supportive function by increasing bioactivity, mechanical stiffness, magnetoelectric properties or providing controlled release of drugs or growth factors.

Shi et al. [171] modified gelatin-methacryloyl (GelMA) with nanohydroxyapatite (HAP) and nonsilicate (SN) and enriched the composite with mesenchymal stem cells (MSCs). Such modification improved osteoinductive properties and provided injectability of the approach by introducing nano-additives. Incorporation of HAP and MSCs was focused on providing biochemical and morphological resemblance to the natural bone tissue. Such an approach was injectable to provide a convenient way of introduction to the defected bone tissue.

A different approach was designed by Chen et al. [172], who developed a self-assembled chitosan-based hydrogel loaded with MgO nanoparticles, which provided sustained release of Mg^2+^ and strengthened hydrogel mechanically. Such a composite was able to withstand the compressive stress similar to that occurring in the bone tissue for 30 days and supported in vitro Ca_3_(PO_4_)_2_ deposition, and at the same time promoted MC3T3-E1 cell proliferation and their differentiation into osteoblasts, and provided bone reconstruction in rats after 12 weeks.

Besides the combination of highly tunable hydrogels with nanoadditives, tissue engineering studies incline toward combined strategies. On the one hand, designed hydrogel/nanoadditive systems serve as scaffolds containing bioactive agents; on the other hand, they deliver drugs to the injured tissue in a controlled way. A great example of such an injectable thermosensitive approach was reported by Taymouri et al. [169]. The chitosan/silk fibroin hydrogel system was modified with dipyridamol/PCL nanoparticles. After appropriate chemical modification, Chitosan provided thermal sensibility, resulting in hydrogels crosslinking at physiological temperature, biochemical properties and osteoconductivity, which is decent from the perspective of bone tissue engineering. Besides low immunogenicity and osteogenerative features, silk fibroin provided c.a 1.5–2.6-fold decreased time of chitosan thermal crosslinking and influenced its mechanical properties. Using dipirydamol provided osteogenesis by activation of A2AR receptors and locking of adenosine escapement, the dipirydamol was loaded into biodegradable PCL nanoparticles to provide better dispersion in the hydrogel matrix as well as to control the release. The MTT test on MG-63 osteoblast-like cells showed increased cell viability and proliferation on the composite material and satisfactory calcium deposition, proving its potential for bone tissue regeneration. However, in vitro drug release tests were conducted for five days only, while the average bone healing, despite using a scaffold, takes between 6 and 12 weeks [173]. In this respect more studies need to be done.

The injectable hydrogel scaffolds/delivery systems are also applied in therapies dedicated to damaged brain and CNS diseases. One of the examples of such an approach is hybrid alginate/chitosan hydrogel loaded with berberine encapsulated inside chitosan (BerNChs) nanoparticles [174]. The hydrogel system provided similarity to the native ECM environment to prevent glial scar formation and adequate mechanical support. Use of nanoparticles provided sustained delivery of Berberine, which plays a crucial role in inhibiting transcription factors responsible for inflammation and carcinogenesis. The stiffness of the obtained hydrogel/nanoparticle system lay within the required CNS range and was 4 kPa. MTT assay on endometrial stem cells (ESCs) showed good cell viability, but most importantly in vivo studies on rats’ spinal cord showed effective delivery of berberine, which provided progressive restoration of limb functions and neuroprotection of injured neural tissue.

Another example of a hydrogel loaded with nanoparticles that combines supportive and delivery functions is a semi-interpenetrating polymer network (semi-IPN) hydrogel consisting of collagen and HA. The hydrogel was additionally loaded with gelatin nanoparticles and chaperone protein (Tat-Hsp70). Gelatin particles improved the viscoelastic properties of the hydrogel system and encapsulated Tat-Hsp70 to ensure its controlled release. The obtained composite provided increased delivery of Tat-Hsp70 compared to delivery of protein alone, resulting in increased protection of neurons, followed by an improved motor function of CD-1 mice [175]. 

In other studies, Zhang [76] et al. designed smart magnetoelectric HA/collagen hydrogel loaded with core/shell Fe_3_O_4_(FO)/BaTiO_3_(BTO) nanoparticles for injured spinal cord regeneration. Such smart material not only consists of biocompatible components but also combines piezoelectricity and magnetic responsivity and provides a native neural ECM environment. The HA/collagen mechanically corresponds to soft neural tissues; the measured Young’s modulus was c.a. 0.4 kPa, due to homogenous nanoparticle dispersion, which provides effective transfer of electric signals. The BTO provides piezoelectricity and FO magnetic properties. The form of the core shell efficiently increases the contact between two phases and hence improves magnetoelectric transduction capacity, T, which corresponds to the native neural tissues. Applying an external magnetic field to cells seeded on a scaffold stimulated PC12 cell transformation into neurons that showed highly elongated morphology compared to cells untreated with a magnetic field. The external magnetic treatment also provided superior expression of proteins specific for neurons (Tuj1, NF and PSD95). Increased expression of PSD95 during electrical stimulation is critical here, since it generates new synapsis formation, crucial from the perspective of effective neural impulse transmission between neural cells. Generally, such an approach has huge potential in enhancing electrical stimulation in vivo and electrical regulation of neurogenesis.

### 5.2. Hydrogels and Electrospun Nanofibers

Another method of hydrogel modification is the addition of electrospun nanofibers. Hydrogel provides the same functions as described in the previous subsection. While electrospun nanofibers strengthen hydrogel mechanically, they offer ECM-mimicking substrates to enhance cell adhesion and differentiation profile [176]. Since electrospun nanofibers could withstand contractile forces, they are attractive support as adhesion sites for cells [177,178]. Above all, the hydrogel/nanofiber composite material provides the environment that entirely mimics the structure of proteoglycans and collagen fibers in the native ECM [179].

Electrospun fibers can be incorporated into the hydrogels using two alternative methods. The first one is stacking layers of nanofibers and hydrogel by lamination and by creating a sandwich model. The method allows to obtain the composite in a precisely controlled and repeatable manner but does not provide injectability of such an approach. The lamination method was used by Zare et al. [180] to form alginate-based hydrogel/gelatin electrospun fibers/Kartogenin-PLGA nanoparticle (KGN-NP) composite. Such a composite served as a scaffold/drug delivery system. Compared to the pure hydrogel, the composite showed c.a. 2.4-fold increased elastic modulus determined from unconfined compressive tests. The in vitro tests on adipocyte mesenchymal stem cells (ADMSCs) assessed using Resazurin assay and Live/Dead staining indicated beneficial cell morphology and viability for composite hydrogel. 

The second method of hydrogel functionalization with electrospun fibers is simpler and more common than the former one. The combination of short electrospun fibers and hydrogel occurs by fibers dispersion in the non-injectable and injectable hydrogel solutions [181]. Maharian et al. functionalized chitosan hydrogel with regenerated cellulose nanofibers (rCLs). The rCLs were obtained by deacetylation of electrospun cellulose acetate fibers. They significantly increased mechanical properties of the injectable hydrogel, provided fibrous structure mimicking collagen fiber network, which is natively present in ECM, and ensured more anchorage site for the mouse calvaria pre-osteoblasts (MC3T3-E1). The incorporation of rCLs to the chitosan not only 1.5-fold increased the Young’s Modulus of chitosan and provided increased nucleation sites for hydroxyapatite, but also CCK-8 assay and confocal laser scanning microscopy (CLSM) imaging showed increased viability, attachment and proliferation of MC3T3-E1 cells [182]. 

In other studies [176], the non-injectable alginate/hyaluronic acid (Alg-g-HA) hydrogel system was modified with poly(lactic acid) (PLA) short electrospun fibers using the same dispersion method. Such an approach served as a scaffold/chondrocyte delivery system for damaged cartilage regeneration. The compression tests on pure hydrogel and hydrogel/fiber systems showed 1.5–1.9-fold increased Young’s modulus after incorporation of PLA fibers. The hydrogel stiffness increased with the higher weight ratio of nanofibers in the composite. The MTT assay confirmed chondrocyte viability after seven days on scaffolds and the non-toxic character of the hydrogel systems. It was also observed that gases and mass transportation occurred adequately throughout the constructs. 

The formation of injectable approaches for tissue engineering through dispersion of nanofibers inside the hydrogel matrix has recently gathered a lot of interest. In this scenario, incorporated electrospun fibers should be fragmented to enable effective injection of the hydrogel system. The aminolysis [162], or motor-driven blade cutting at −80 °C [183], are just a few examples of electrospun nanofiber fragmentation. Injectable hydrogels are especially important while material should be introduced into inaccessible areas in the tissue in a short time. The great example of such an area is neural tissue. Hsieh et al. [184] formed a smart thermosensitive/shear-thinning hyaluronic acid/methylcellulose (HAMC) hydrogel, which was subsequently loaded alternatively with collagen and poly(ε-caprolactone-co-D,L-lactide (P(CL:DLLA))). Such an approach served as a scaffold/cell delivery system for regeneration of spinal cord. The CellTiter-Glo^®^ Luminescent Cell Viability assay and Live/Dead assay showed the best survival of neural stem/progenitor cells (NSPCs) and was visible for the HAMC/P(CL:DLLA system. Additionally, on this material NSPCs evidently differentiated into neural cells and oligodendrocytes, which are responsible for myelin formation, and inhibit the degeneration of axons.

Similar research was conducted by Ghaderinejad et al. [185]. The injectable alginate hydrogel system was loaded with magnetic short PCL/superparamagnetic iron oxide nanoparticle (SPION) electrospun fibers. Such an approach served as a non-invasive scaffold for neural regeneration. Besides mechanical support and providing fibrous structure, concerning the presence of magnetic particles, the fibers were aligned without aggregation within the hydrogel system after in situ crosslinking. The unique architecture of hydrogel/short fiber systems promoted extended and flattened olfactory ecto-mesenchymal stem cell (OE-MSC) morphology, provided their increased proliferation and showed great potential concerning their differentiation into dopaminergic neurons

## 6. Conclusions and Future Perspectives

There is continuing dynamic development in tissue engineering toward new scientific trends for curing, diagnosing and preventing diseases at the nano- and micro-levels. Innovations in these materials concerning fabrication processes allow them to produce implants with good performance. 

The newest trend in designing scaffolds for bone/cartilage and CNS tissue engineering is combining at least two different materials, e.g., injectable smart hydrogels with nano-additives. Such modifications overcome the limitations of materials occurring alone, might increase stiffness of hydrogels or provide biocompatibility to biologically inert materials. Additionally, they might provide yet undescribed properties such as magnetoelectricity or injection ability of electrospun nanofibers.

An attractive solution is also combining at least two fields of tissue engineering, i.e., drug and cell delivery system/scaffold, to make a synergic effect which is very promising from the perspective of tissue engineering, particularly for bone/cartilage and neural regeneration. Such an attitude allows overcoming fundamental limitations of currently used clinical materials dedicated to bone/cartilage and neural treatments.

Besides primary characterization, future studies should focus on designing multicomponent materials that will not only mimic ECM, deliver drugs and growth factors or provide electrical stimulation, but will also fit the in vivo mechanisms and mechanobiology of bone/cartilage and neural regeneration [186]. It is also crucial to monitor the use of scaffolds in clinical conditions for a long time after implantation. Such studies will evaluate the safety and effectiveness of the used material, evaluating its real relevance as a scaffold for bone/cartilage and neural tissue engineering application.

## Figures and Tables

**Table 1:** List of commercially used hydrogels for tissue engineering applications.

Trade Name	Material	Biomedical Application	Property	Ref.
EUFLEXXA^®^	HA	Knee osteoarthritis	Artificial knee synovial fluid	[111]
HyStem™	synthetic HA modified with functional thiol-groups	Cartilage tissue engineering	Scaffold for cartilage regeneration	[112]
Biogelx	di- or tri-peptides modified at the N-terminus with the aromatic structure (i.e., fluorenylmethoxycarbonyl, Fmoc)	Tissue engineering	Scaffold for cell culturing/regeneration	[113]
Collagraft^®^	Hydroxyapatite/Tricalcium phosphate/Collagen	Bone tissue engineering	Bone graft	[114]
INFUSE^®^	Collagen	Bone tissue engineering	Bone graft	[98]
Hyalgan^®^	HA	Bone/cartilage tissue engineering	support for the knees/joints in osteoarthritis	[115]
Hyalubrix^®^	HA	Bone/cartilage tissue engineering	support for the knees/joints in osteoarthritis	[115]
Mebiol^®^	PNIPAAm-PEG	Neural tissue engineering	Scaffold/drug delivery to brain tumors	[116]
Poloxamer	triblock copolymers of poly(propylene oxide) and poly(ethylene oxide) (PPO-PEO)	Articular cartilage/	Thermoresponsive scaffold for cell regeneration	[117]
Pluronics^®^	block copolymers of PPO-PEO	Bone/neural tissue engineering	Scaffold for cell regeneration	[118,119]
Pura Matrix		Neural tissue engineering	Scaffold for cell regeneration	[106]
Corning^®^ Matrigel^®^	laminin, collagen IV, entactin, nidogen and heparan sulfate proteoglycans. Growth factors such as TGF-β, basic fibroblast growth factor, insulin-like growth factor-1 and tissue plasminogen activator	Neural tissue engineering	Scaffold for cell regeneration	[106]

**Table 2:** Selected examples of commercially used scaffolds for bone/cartilage and neural tissue engineering applications from polymers.

Trade Name	Polymer	Biomedical Application	Property	Ref.
Dexon	PGA	Biodegradable synthetic suture	Tissue regeneration, PLLA high-strength fibers	[132]
Monacryl	PDLLA-CL	Monofilament suture	Fibers less stiff	[133]
Pins	PDS	Fixation screws in small bones	Orthopaedic applications	[134]
Maxon	PCLTMC	Flexible suture materials	multiblock	[135]
Acufex	PCLTMC and PGCL	Orthopaedic screws	multiblock	[136]
No trade name	PHBHV	Bone pins, drug delivery, plates	piezoelectric properties	[137]
Dacron	PLLA	vessel	Fiber-based devices	[138]
Synvisc, Orthovisc	HAP	Relieve pain, improve joint mobility	Synovial fluid substitute	[139]
Polynova	LDI-based PU	Orthopedic applications	Injectable, high mechanical properties	[140]
NeuroMatrix™	Collagen	Neural applications	Tubular matrix	[141]
Neuroflex™	Collagen	Neural applications	Protect axonal growth across nerve gap	[142]
Neuragen^®^	Collagen	Neural applications	Nerve guide	[143, 144]
Neurolac™	PLA-PCL	Recovery of sensory nerve function	Nerve conduit	[145]
NeuroTube^®^	PGA	Peripheral nerve gaps	Nerve conduit	[146]
SaluTunnel	Salubria, non-biodegradable	Peripheral nerve gaps	Nerve conduit	[147]

## Data Availability

Not applicable.

## References

[1] Mo X., Sun B., Wu T., Li D., Ding B., Wang X., Yu J. (2019). Electrospun nanofibers for tissue engineering. Electrospinning: Nanofabrication and Applications.

[2] Li X., Chen S., Li J., Wang X., Zhang J., Kawazoe N., Chen G. (2016). 3D culture of chondrocytes in gelatin hydrogels with different stiffness. Polymers.

[3] Wang X., Ding B., Li B. (2013). Biomimetic electrospun nanofibrous structures for tissue engineering. Mater. Today.

[4] Caliari S.R., Burdick J.A. (2016). A practical guide to hydrogels for cell culture. Nat. Methods.

[5] Advincula R.C., Dizon J.R.C., Caldona E.B., Viers R.A., Siacor F.D.C., Maalihan R.D., Espera A.H. (2021). On the progress of 3D-printed hydrogels for tissue engineering. MRS Commun..

[6] Hutmacher D.W. (2000). Scaffolds in tissue engineering bone and cartilage. Biomaterials.

[7] Zaszczyńska A., Moczulska-Heljak M., Gradys A., Sajkiewicz P. (2021). Advances in 3D Printing for Tissue Engineering. Materials.

[8] Vakiel P., Shekarforoush M., Dennison C.R., Scott M., Muench G., Hart D.A., Shrive N.G. (2021). Mapping stresses on the Tibial plateau cartilage in an ovine model using in-vivo gait kinematics. Ann. Biomed. Eng..

[9] Goganau I., Sandner B., Weidner N., Fouad K., Blesch A. (2018). Depolarization and electrical stimulation enhance in vitro and in vivo sensory axon growth after spinal cord injury. Exp. Neurol..

[10] Baldwin P., Li D.J., Auston D.A., Mir H.S., Yoon R.S., Koval K.J. (2019). Autograft, Allograft, and Bone Graft Substitutes: Clinical Evidence and Indications for Use in the Setting of Orthopaedic Trauma Surgery. J. Orthop. Trauma.

[11] Borgstrom F., Karlsson L., Ortsater G., Norton N., Halbout P., Cooper C., Lorentzon M., McCloskey E.V., Harvey N.C., Javaid M.K. (2020). Fragility Fractures in Europe: Burden, Management and Opportunities. Arch. Osteoporos..

[12] Lo J., Chan L., Flynn S. (2021). A Systematic Review of the Incidence, Prevalence, Costs, and Activity and Work Limitations of Amputation, Osteoarthritis, Rheumatoid Arthritis, Back Pain, Multiple Sclerosis, Spinal Cord Injury, Stroke, and Traumatic Brain Injury in the United States: A 2019 Update. Arch. Phys. Med. Rehabil..

[13] James S.L., Abate D., Abate K.H., Abay S.M., Abbafati C., Abbasi N., Abbastabar H., Abd-Allah F., Abdela J., Abdelalim A. (2018). Global, Regional, and National Incidence, Prevalence, and Years Lived with Disability for 354 Diseases and Injuries for 195 Countries and Territories, 1990–2017: A Systematic Analysis for the Global Burden of Disease Study 2017. Lancet.

[14] Hunter D.J., Schofield D., Callander E. (2014). The Individual and Socioeconomic Impact of Osteoarthritis. Nat. Rev. Rheumatol..

[15] World Health Organization Dementia. https://www.who.int/news-room/fact-sheets/detail/dementia.

[16] Blamire A.M. (2018). MR Approaches in Neurodegenerative Disorders. Prog. Nucl. Magn. Reson. Spectrosc..

[17] Fish P.V., Steadman D., Bayle E.D., Whiting P. (2019). New Approaches for the Treatment of Alzheimer’s Disease. Bioorganic Med. Chem. Lett..

[18] James S.L., Theadom A., Ellenbogen R.G., Bannick M.S., Montjoy-Venning W., Lucchesi L.R., Abbasi N., Abdulkader R., Abraha H.N., Adsuar J.C. (2019). Global, Regional, and National Burden of Traumatic Brain Injury and Spinal Cord Injury, 1990–2016: A Systematic Analysis for the Global Burden of Disease Study 2016. Lancet Neurol..

[19] Williams D.F. (2008). On the Mechanisms of Biocompatibility. Biomaterials.

[20] Mariani E., Lisignoli G., Borzì R.M., Pulsatelli L. (2019). Biomaterials: Foreign Bodies or Tuners for the Immune Response?. Int. J. Mol. Sci..

[21] Morris A.H., Stamer D.K., Kyriakides T.R. (2017). The Host Response to Naturally-Derived Extracellular Matrix Biomaterials. Semin. Immunol..

[22] Klopfleisch R., Jung F. (2017). The Pathology of the Foreign Body Reaction against Biomaterials: Foreign Body Reaction to Biomaterials. J. Biomed. Mater. Res..

[23] Vishwakarma A., Bhise N.S., Evangelista M.B., Rouwkema J., Dokmeci M.R., Ghaemmaghami A.M., Vrana N.E., Khademhosseini A. (2016). Engineering Immunomodulatory Biomaterials To Tune the Inflammatory Response. Trends Biotechnol..

[24] Li H., Shen S., Fu H., Wang Z., Li X., Sui X., Yuan M., Liu S., Wang G., Guo Q. (2019). Immunomodulatory Functions of Mesenchymal Stem Cells in Tissue Engineering. Stem Cells Int..

[25] He J., Chen G., Liu M., Xu Z., Chen H., Yang L., Lv Y. (2020). Scaffold Strategies for Modulating Immune Microenvironment during Bone Regeneration. Mater. Sci. Eng. C.

[26] Kulkarni V., Butte K., Rathod S. (2012). Natural Polymers- A Comprehensive Review. Int. J. Pharm. Biomed. Res..

[27] Dawson E., Mapili G., Erickson K., Taqvi S., Roy K. (2008). Biomaterials for Stem Cell Differentiation. Adv. Drug Deliv. Rev..

[28] Reddy N., Reddy R., Jiang Q. (2015). Crosslinking Biopolymers for Biomedical Applications. Trends Biotechnol..

[29] Casali D.M., Yost M.J., Matthews M.A. (2018). Eliminating Glutaraldehyde from Crosslinked Collagen Films Using Supercritical CO_2_. J. Biomed. Mater. Res..

[30] Karoyo A.H., Wilson L.D. (2021). A Review on the Design and Hydration Properties of Natural Polymer-Based Hydrogels. Materials.

[31] Nezhad-Mokhtari P., Ghorbani M., Roshangar L., Soleimani Rad J. (2019). Chemical Gelling of Hydrogels-Based Biological Macromolecules for Tissue Engineering: Photo- and Enzymatic-Crosslinking Methods. Int. J. Biol..

[32] Ehrmann A. (2021). Non-Toxic Crosslinking of Electrospun Gelatin Nanofibers for Tissue Engineering and Biomedicine—A Review. Polymers.

[33] Karakus G., Zengin H.B., Polat Z.A., Yenidunya A.F., Aydin S. (2013). Cytotoxicity of Three Maleic Anhydride Copolymers and Common Solvents Used for Polymer Solvation. Polym. Bull..

[34] Zhang B., Yan X., He H.-W., Yu M., Ning X., Long Y.-Z. (2017). Solvent-Free Electrospinning: Opportunities and Challenges. Polym. Chem..

[35] Farris A.L., Rindone A.N., Grayson W.L. (2016). Oxygen Delivering Biomaterials for Tissue Engineering. J. Mater. Chem. B.

[36] Ejtehadifar M., Shamsasenjan K., Movassaghpour A., Akbarzadehlaleh P., Abbasi P., Molaeipour Z., Saleh M. (2015). The Effect of Hypoxia on Mesenchymal Stem Cell Biology. Adv. Pharm. Bull..

[37] Mohyeldin A., Garzon-Muvdi T., Quinones-Hinojosa A. (2010). Oxygen in Stem Cell Biology: A Critical Component of the Stem Cell. Niche Cell Stem Cell.

[38] Di Mattia M., Mauro A., Citeroni M.R., Dufrusine B., Peserico A., Russo V., Berardinelli P., Dainese E., Cimini A., Barboni B. (2021). Insight into Hypoxia Stemness Control. Cells.

[39] Von der Mark K., Park J. (2013). Engineering Biocompatible Implant Surfaces. Prog. Mater. Sci..

[40] Ferrari M., Cirisano F., Morán M.C. (2019). Mammalian Cell Behavior on Hydrophobic Substrates: Influence of Surface Properties. Colloids Interfaces.

[41] Metwally S., Stachewicz U. (2019). Surface Potential and Charges Impact on Cell Responses on Biomaterials Interfaces for Medical Applications. Mater. Sci. Eng. C.

[42] Yue S., He H., Li B., Hou T. (2020). Hydrogel as a Biomaterial for Bone Tissue Engineering: A Review. Nanomaterials.

[43] Denry I., Kuhn L.T. (2016). Design and Characterization of Calcium Phosphate Ceramic Scaffolds for Bone Tissue Engineering. Dent. Mater..

[44] Li X., Wang L., Fan Y., Feng Q., Cui F.-Z., Watari F. (2013). Nanostructured Scaffolds for Bone Tissue Engineering. J. Biomed. Mater. Res..

[45] Loh Q.L., Choong C. (2013). Three-Dimensional Scaffolds for Tissue Engineering Applications: Role of Porosity and Pore Size. Tissue Eng. Part B Rev..

[46] Diaz-Rodriguez P., Garcia-Trinanes P., Lopez M.E., Santovena A., Landin M. (2018). Mineralized alginate hydrogels using marine carbonates for bone tissue engineering applications. Carbohydr. Polym..

[47] Ko F.C., Sumner D.R. (2020). How faithfully does intramembranous bone regeneration recapitulate embryonic skeletal development?. Dev. Dyn..

[48] Aghajanian P., Mohan S. (2018). The art of building bone: Emerging role of chondrocyte-to-osteoblast transdifferentiation in endochondral ossification. Bone Res..

[49] Majidinia M., Aghazadeh J., Jahanban-Esfahlani R., Yousefi B. (2018). The roles of Wnt/β-catenin pathway in tissue development and regenerative medicine. J. Cell. Physiol..

[50] Rachit A., Garcia A.J. (2015). Biomaterial strategies for engineering implants for enhanced osseointegration and bone repair. Adv. Drug Deliv. Rev..

[51] Carletti E., Motta A., Migliaresi C. (2011). Scaffolds for tissue engineering and 3D cell culture. 3D Cell Culture.

[52] Amini A.R., Laurencin C.T., Nukavarapu S.P. (2012). Bone Tissue Engineering: Recent Advances and Challenges. Crit. Rev. Biomed. Eng..

[53] Chatterjee K., Lin-Gibson S., Wallace W.E., Parekh S.H., Lee Y.J., Cicerone M.T., Young M.F., Simon C.G. (2010). The Effect of 3D Hydrogel Scaffold Modulus on Osteoblast Differentiation and Mineralization Revealed by Combinatorial Screening. Biomaterials.

[54] Poumarat G., Squire P. (1993). Comparison of mechanical properties of human, bovine bone and a new processed bone xenograft. Biomaterials.

[55] Hofmann S., Garcia-Fuentes M., Eberli D. (2011). Bioactive Scaffolds for the Controlled Formation of Complex Skeletal Tissues. Regenerative Medicine and Tissue Engineering—Cells and Biomaterials.

[56] Chandran V., Coppola G., Nawabi H., Omura T., Versano R., Huebner E.A., Zhang A., Costigan M., Yekkirala A., Barrett L. (2016). A Systems-Level Analysis of the Peripheral Nerve Intrinsic Axonal Growth. Program. Neuron.

[57] Burda J.E., Sofroniew M.V. (2014). Reactive Gliosis and the Multicellular Response to CNS Damage and Disease. Neuron.

[58] Li X., Liu D., Xiao Z., Zhao Y., Han S., Chen B., Dai J. (2019). Scaffold-Facilitated Locomotor Improvement Post Complete Spinal Cord Injury: Motor Axon Regeneration versus Endogenous Neuronal Relay Formation. Biomaterials.

[59] Engler A.J., Sen S., Sweeney H.L., Discher D.E. (2006). Matrix Elasticity Directs Stem Cell Lineage Specification. Cell.

[60] Saha K., Keung A.J., Irwin E.F., Li Y., Little L., Schaffer D.V., Healy K.E. (2008). Substrate Modulus Directs Neural Stem Cell Behavior. Biophys. J..

[61] Kothapalli C., Mahajan G., Farrell K. (2020). Substrate Stiffness Induced Mechanotransduction Regulates Temporal Evolution of Human Fetal Neural Progenitor Cell Phenotype, Differentiation, and Biomechanics. Biomater. Sci..

[62] Simitzi C., Ranella A., Stratakis E. (2017). Controlling the Morphology and Outgrowth of Nerve and Neuroglial Cells: The Effect of Surface Topography. Acta Biomater..

[63] Niemczyk B., Sajkiewicz P., Kolbuk D. (2018). Injectable Hydrogels as Novel Materials for Central Nervous System Regeneration. J. Neural Eng..

[64] Dhoot N.O., Tobias C.A., Fischer I., Wheatley M.A. (2004). Peptide-Modified Alginate Surfaces as a Growth Permissive Substrate for Neurite Outgrowth. J. Biomed. Mater. Res..

[65] Shaw D., Shoichet M.S. (2003). Toward Spinal Cord Injury Repair Strategies: Peptide Surface Modification of Expanded Poly(Tetrafluoroethylene) Fibers for Guided Neurite Outgrowth In Vitro. J. Craniofac. Surg..

[66] Adak A., Das G., Barman S., Mohapatra S., Bhunia D., Jana B., Ghosh S. (2017). Biodegradable Neuro-Compatible Peptide Hydrogel Promotes Neurite Outgrowth, Shows Significant Neuroprotection, and Delivers Anti-Alzheimer Drug. ACS Appl. Mater. Interfaces.

[67] Ren Y.-J., Zhang H., Huang H., Wang X.-M., Zhou Z.-Y., Cui F.-Z., An Y.-H. (2009). In Vitro Behavior of Neural Stem Cells in Response to Different Chemical Functional Groups. Biomaterials.

[68] Li L., El-Hayek Y.H., Liu B., Chen Y., Gomez E., Wu X., Ning K., Li L., Chang N., Zhang L. (2008). Direct-Current Electrical Field Guides Neuronal Stem/Progenitor Cell Migration. Stem Cells.

[69] Willand M.P., Nguyen M.-A., Borschel G.H., Gordon T. (2016). Electrical Stimulation to Promote Peripheral Nerve Regeneration. Neurorehabil. Neural Repair.

[70] Uz M., Mallapragada S.K. (2019). Conductive Polymers and Hydrogels for Neural Tissue Engineering. J. Indian. Inst. Sci..

[71] Farokhi M., Mottaghitalab F., Saeb M.R., Shojaei S., Zarrin N.K., Thomas S., Ramakrishna S. (2021). Conductive Biomaterials as Substrates for Neural Stem Cells Differentiation towards Neuronal Lineage Cells. Macromol. Biosci..

[72] Perez-Garnes M., Barcia J.A., Gomez-Pinedo U., Monleon Pradas M., Valles-Lluch A., Eberli D. (2014). Materials for Central Nervous System Tissue Engineering. Cells and Biomaterials in Regenerative Medicine.

[73] Yu L.M.Y., Leipzig N.D., Shoichet M.S. (2008). Promoting Neuron Adhesion and Growth. Mater. Today.

[74] Lis A., Szarek D., Laska J. (2013). Biomaterials engineering strategies for spinal cord regeneration: State of the art. Polim. Med..

[75] Akhtari M., Emin D., Ellingson B.M., Woodworth D., Frew A., Mathern G.W. (2016). Measuring the local electrical conductivity of human brain tissue. J. Appl. Phys..

[76] Zhang Y., Chen S., Xiao Z., Liu X., Wu C., Wu K., Fan H. (2021). Magnetoelectric Nanoparticles Incorporated Biomimetic Matrix for Wireless Electrical Stimulation and Nerve Regeneration. Adv. Healthc. Mater..

[77] Balint R., Cassidy N.J., Cartmell S.H. (2014). Conductive polymers: Towards a smart biomaterial for tissue engineering. Acta Biomater..

[78] Niemczyk-Soczynska B., Gradys A., Kolbuk D., Krzton-Maziopa A., Sajkiewicz P. (2019). Crosslinking kinetics of methylcellulose qqueous solution and its potential as a scaffold for tissue engineering. Polymers.

[79] Catoira M.C., Fusaro L., Di Francesco D., Ramella M., Boccafoschi F. (2019). Overview of natural hydrogels for regenerative medicine applications. J. Mater. Sci. Mater. Med..

[80] Singh M.R., Patel S., Singh D., Grumezesc A. (2016). Natural polymer-based hydrogels as scaffolds for tissue engineering. Nanobiomaterials in Soft Tissue Engineering.

[81] Madduma-Bandarage U.S., Madihally S.V. (2021). Synthetic hydrogels: Synthesis, novel trends, and applications. J. Appl. Polym. Sci..

[82] Cimen Z., Babadag S., Odabas S., Altuntas S., Demirel G., Demirel G.B. (2021). Injectable and Self-Healable pH-Responsive Gelatin–PEG/Laponite Hybrid Hydrogels as Long-Acting Implants for Local Cancer Treatment. ACS Appl. Polym..

[83] He Y., Wang F., Wang X., Zhang J., Wang D., Huang X. (2021). A photocurable hybrid chitosan/acrylamide bioink for DLP based 3D bioprinting. Mater. Des.

[84] Zou X., Zhao X., Ye L. (2015). Synthesis of cationic chitosan hydrogel and its controlled glucose-responsive drug release behavior. Chem. Eng. J..

[85] Yamanlar S., Sant S., Boudou T., Picart C., Khademhosseini A. (2011). Surface functionalization of hyaluronic acid hydrogels by polyelectrolyte multilayer films. Biomaterials.

[86] Nakajima O., Mizoguchi H., Hashimoto Y., Iwasaki S. (1992). Non-ionic water-soluble dextran-coupled tetraphenylporphyrin derivatives. J. Am. Chem. Soc..

[87] Loh E.Y.X., Fauzi M.B., Ng M.H., Ng P.Y., Ng S.F., Amin M.C.I.M. (2020). Insight into delivery of dermal fibroblast by non-biodegradable bacterial nanocellulose composite hydrogel on wound healing. Int. J. Biol. Macromol..

[88] Aswathy S.H., Narendrakumar U., Manjubala I. (2020). Commercial hydrogels for biomedical applications. Heliyon.

[89] Glickman R.D., Onorato M., Campos M.M., O’Boyle M.P., Singh R.K., Zarembinski T.I., Nasonkin I.O. (2021). Intraocular Injection of HyStem Hydrogel Is Tolerated Well in the Rabbit Eye. J. Ocul. Pharmacol. Ther..

[90] Hocevar S.E., Liu L., Duncan R.K. (2021). Matrigel is required for efficient differentiation of isolated, stem cell-derived otic vesicles into inner ear organoids. Stem Cell Res..

[91] Strauß S., Meutelet R., Radosevic L., Gretzinger S., Hubbuch J. (2021). Image analysis as PAT-Tool for use in extrusion-based bioprinting. Bioprinting.

[92] Ping J., Qi L., Wang Q., Liu S., Jiang Y., Yu L., Hu Q. (2021). An integrated liquid crystal sensing device assisted by the surfactant-embedded smart hydrogel. Biosens. Bioelectron..

[93] Esmaeely Neisiany R., Enayati M.S., Sajkiewicz P., Pahlevanneshan Z., Ramakrishna S. (2020). Insight into the current directions in functionalized nanocomposite hydrogels. Front. Mater. Sci..

[94] Katoh S., Yoshioka H., Senthilkumar R., Preethy S., Abraham S.J. (2021). Enhanced expression of hyaluronic acid in osteoarthritis-affected knee-cartilage chondrocytes during three-dimensional in vitro culture in a hyaluronic-acid-retaining polymer scaffold. Knee.

[95] Li Q., Wang Q., Wang O., Shao K., Lin H., Lei Y. (2018). A simple and scalable hydrogel-based system for culturing protein-producing cells. PLoS ONE.

[96] YunXiu L., YuZhen W., MingZhu J., Xuan T., Feng H., XinZhi Y. (2021). Organoid culture of mouse fallopian tube epithelial stem cells with a thermo-reversible gelation polymer. Tissue Cell.

[97] Gupta D., Tator C.H., Shoichet M.S. (2006). Fast-gelling injectable blend of hyaluronan and methylcellulose for intrathecal, localized delivery to the injured spinal cord. Biomaterials.

[98] Mandal A., Clegg J.R., Anselmo A.C., Mitragotri S. (2020). Hydrogels in the clinic. Bioeng. Transl. Med..

[99] Zhang X., Tan B., Wu Y., Zhang M., Liao J. (2021). A Review on Hydrogels with Photothermal Effect in Wound Healing and Bone Tissue Engineering. Polymers.

[100] Kirschner C.M., Anseth K.S. (2013). Hydrogels in healthcare: From static to dynamic material microenvironments. Acta Mater..

[101] Skardal A., Zhang J., McCoard L., Xu X., Oottamasathien S., Prestwich G.D. (2010). Photocrosslinkable hyaluronan-gelatin hydrogels for two-step bioprinting. Tissue Eng. Part A.

[102] Yu C., Gao H., Li Q., Cao X. (2020). Injectable dual cross-linked adhesive hyaluronic acid multifunctional hydrogel scaffolds for potential applications in cartilage repair. Polym. Chem..

[103] Wu J., Chen Q., Deng C., Xu B., Zhang Z., Yang Y., Lu T. (2020). Exquisite design of injectable hydrogels in cartilage repair. Theranostics.

[104] Bellotti E., Schilling A.L., Little S.R., Decuzzi P. (2020). Injectable thermoresponsive hydrogels as drug delivery system for the treatment of central nervous system disorders: A review. J. Control Release.

[105] Sun H., Zhang L., Cheng W., Hao F., Zhou L., Li Q. (2021). Injectable Hydrogels in Repairing Central Nervous System Injuries. Adv. Mater. Sci. Eng..

[106] Thonhoff J.R., Lou D.I., Jordan P.M., Zhao X., Wu P. (2008). Compatibility of human fetal neural stem cells with hydrogel biomaterials in vitro. Brain Res..

[107] Rinoldi C., Lanzi M., Fiorelli R., Nakielski P., Zembrzycki K., Kowalewski T., Pierini F. (2021). Three-Dimensional Printable Conductive Semi-Interpenetrating Polymer Network Hydrogel for Neural Tissue Applications. Biomacromolecules.

[108] Grover G.N., Braden R.L., Christman K.L. (2013). Oxime cross-linked injectable hydrogels for catheter delivery. Adv. Mater..

[109] Hu M., Yang J., Xu J. (2021). Structural and biological investigation of chitosan/hyaluronic acid with silanized-hydroxypropyl methylcellulose as an injectable reinforced interpenetrating network hydrogel for cartilage tissue engineering. Drug Deliv..

[110] Yu C., Yao F., Li J. (2021). Rational design of injectable conducting polymer-based hydrogels for tissue engineering. Acta Biomater..

[111] Nicholls M., Manjoo A., Shaw P., Niazi F., Rosen J. (2018). A Comparison Between Rheological Properties of Intra-articular Hyaluronic Acid Preparations and Reported Human Synovial Fluid. Adv. Ther..

[112] Ylarinne J.H., Qu C., Lammi M.J. (2017). Scaffold-free approach produces neocartilage tissue of similar quality as the use of HyStem™ and Hydromatrix™ scaffolds. J. Mater. Sci. Mater. Med..

[113] Harper M.M., Connolly M.L., Goldie L., Irvine E.J., Shaw J.E., Jayawarna V., Ulijn R.V. (2018). Biogelx: Cell Culture on Self-Assembling Peptide Gels. Peptide Self-Assembly.

[114] Cornell C.N., Lane J.M., Chapman M., Merkow R., Seligson D., Henry S., Vincent K. (1991). Multicenter trial of Collagraft as bone graft substitute. J. Orthop. Trauma.

[115] Alnasser S., AlHussain F., Asiri H., Almutairi A., Alsanawi H., Altamimi A.A., AlRuthia Y. (2021). Orthopedic Surgeons’ Views of Hyaluronic Acid Formulations in the Management of Knee Osteoarthritis: A Questionnaire-Based Cross-Sectional Study. Medicina.

[116] Ozeki T., Kaneko D., Hashizawa K., Imai Y., Tagami T., Okada H. (2012). Improvement of survival in C6 rat glioma model by a sustained drug release from localized PLGA microspheres in a thermoreversible hydrogel. Int. J. Pharm..

[117] Russo E., Villa C. (2019). Poloxamer hydrogels for biomedical applications. Pharmaceutics.

[118] Lippens E., Vertenten G., Gironès J., Declercq H., Saunders J., Luyten J., Cornelissen M. (2010). Evaluation of Bone Regeneration with an Injectable, In Situ Polymerizable Pluronic^®^F127 Hydrogel Derivative Combined with Autologous Mesenchymal Stem Cells in a Goat Tibia Defect Model. Tissue Eng. A.

[119] Vacanti M.P., Leonard J.L., Vacanti C., Dore B., Bonassar L. (2001). Tissue-engineered spinal cord. Transplant. Proc..

[120] Zaszczynska A., Sajkiewicz P., Gradys A. (2020). Piezoelectric scaffolds as smart materials for neural tissue engineering. Polymers.

[121] Keshvardoostchokami M., Majidi S.S., Huo P., Ramachandran R., Chen M., Liu B. (2021). Electrospun Nanofibers of Natural and Synthetic Polymers as Artificial Extracellular Matrix for Tissue Engineering. Nanomater.

[122] Chen J., Xu J., Wang A., Zheng M. (2009). Scaffolds for tendon and ligament repair: Review of the efficacy of commercial products. Expert Rev. Med. Devices.

[123] Zaszczynska A., Gradys A., Sajkiewicz P. (2020). Progress in the applications of smart piezoelectric materials for medical devices. Polymers.

[124] Singhal A.R., Agrawal C.M., Athanasiou K.A. (1996). Salient degradation features of a 50:50 PLA/PGA scaffold for tissue engineering. Tissue Eng..

[125] Prasadh S., Wong R.C.W. (2018). Unraveling the mechanical strength of biomaterials used as a bone scaffold in oral and maxillofacial defects. Oral Sci. Int..

[126] Barber F.A., McGarry J.E., Herbert M.A., Anderson R.B. (2008). A biomechanical study of Achilles tendon repair augmentation using GraftJacket matrix. Foot Ankle Int..

[127] Guidoin M.F., Marois Y., Bejui J., Poddevin N., King M.W., Guidoin R. (2000). Analysis of retrieved polymer fiber based replacements for the ACL. Biomaterials.

[128] Valentin J.E., Badylak J.S., McCabe G.P., Badylak S.F. (2006). Extracellular matrix bioscaffolds for orthopaedic applications: A comparative histologic study. JBJS.

[129] Debnath U.K., Fairclough J.A., Williams R.L. (2004). Long-term local effects of carbon fibre in the knee. Knee.

[130] Trieb K., Blahovec H., Brand G., Sabeti M., Dominkus M., Kotz R. (2004). In vivo and in vitro cellular ingrowthinto a new generation of artificial ligaments. Eur. Surg. Res..

[131] Lavoie P., Fletcher J., Duval N. (2000). Patient satisfaction needs as related to knee stability and objective findings after ACL reconstruction using the LARS artificial ligament. Knee.

[132] Terasaka S., Iwasaki Y., Shinya N., Uchida T. (2006). Fibrin glue and polyglycolic acid nonwoven fabric as a biocompatible dural substitute. Oper. Neurosurg..

[133] Maurus P.B., Kaeding C.C. (2004). Bioabsorbable implant material review. Oper. Tech. Sports Med..

[134] Rokkanen P.U., Bostman O., Hirvensalo E., Mäkelä E.A., Partio E.K., Patiala H., Tormala P. (2000). Bioabsorbable fixation in orthopaedic surgery and traumatology. Biomaterials.

[135] Doppalapudi S., Jain A., Khan W., Domb A.J. (2014). Biodegradable polymers—An overview. Polym. Adv. Technol..

[136] Thadepalli S. (2021). Review of multifarious applications of polymers in medical and health care textiles. Mater. Today Proc..

[137] Pouton C.W., Akhtar S. (1996). Biosynthetic polyhydroxyalkanoates and their potential in drug delivery. Adv. Drug Deliv. Rev..

[138] Zilberman M., Nelson K.D., Eberhart R.C. (2005). Mechanical properties and in vitro degradation of bioresorbable fibers and expandable fiber-based stents. J. Biomed. Mater. Res. B Appl. Biomater..

[139] Kato Y., Nakamura S., Nishimura M. (2006). Beneficial actions of hyaluronan (HA) on arthritic joints: Effects of molecular weight of HA on elasticity of cartilage matrix. Biorheology.

[140] Bonzani I.C., Adhikari R., Houshyar S., Mayadunne R., Gunatillake P., Stevens M.M. (2007). Synthesis of two-component injectable polyurethanes for bone tissue engineering. Biomaterials.

[141] Tian L., Prabhakaran M.P., Ramakrishna S. (2015). Strategies for regeneration of components of nervous system: Scaffolds, cells and biomolecules. Regen. Biomater..

[142] Shahriari D., Shibayama M., Lynam D.A., Wolf K.J., Kubota G., Koffler J.Y., Tuszynski M.H., Campana W.M., Jeff S., Sakamoto J.S. (2017). Peripheral nerve growth within a hydrogel microchannel scaffold supported by a kink-resistant conduit. J. Biomed. Mater. Res. A.

[143] Lohmeyer J.A., Siemers F., Machens H.G., Mailänder P. (2009). The clinical use of artificial nerve conduits for digital nerve repair: A prospective cohort study and literature review. J. Reconstr. Microsurg..

[144] Archibald. S., Shefner. J., Krarup C., Madison R.D. (1995). Monkey median nerve repaired by nerve graft or collagen nerve guide tube. J. Neurosci..

[145] Bertleff M.J., Meek M.F., Nicolai J.P. (2005). A prospective clinical evaluation of biodegradable neurolac nerve guides for sensory nerve repair in the hand. J. Hand Surg..

[146] Donoghoe N., Rosson G.D., Dellon A.L. (2007). Reconstruction of the human median nerve in the forearm with the Neurotube™. Microsurgery.

[147] Sensharma P., Madhumathi G., Jayant R.D., Jaiswal A.K. (2017). Biomaterials and cells for neural tissue engineering: Current choices. Mater. Sci. Eng. C.

[148] Deal D.N., Griffin J.W., Hogan M.V. (2012). Nerve conduits for nerve repair or reconstruction. J. Am. Acad. Orthop. Surg..

[149] di Summa P.G., Kingham P.J., Campisi C.C., Raffoul W., Kalbermatten D.F. (2014). Collagen (NeuraGen^®^) nerve conduits and stem cells for peripheral nerve gap repair. Neurosci. Lett..

[150] Du J., Chen H., Qing L., Yang X., Jia X. (2018). Biomimetic neural scaffolds: A crucial step towards optimal peripheral nerve regeneration. Biomater. Sci..

[151] Mu Y., Wu F., Lu Y., Wei L., Yuan W. (2014). Progress of electrospun fibers as nerve conduits for neural tissue repair. Nanomedicine.

[152] Gerth D.J., Tashiro J., Thaller S.R. (2015). Clinical outcomes for Conduits and Scaffolds in peripheral nerve repair. World J. Clin. Cases WJCC.

[153] Huang C., Zeng P., Yang S., Shao Y., Liu Y. (2016). Water reclamation and reuse. Water Environ. Res..

[154] Gaudin R., Knipfer C., Henningsen A., Smeets R., Heiland M., Hadlock T. (2016). Approaches to peripheral nerve repair: Generations of biomaterial conduits yielding to replacing autologous nerve grafts in craniomaxillofacial surgery. BioMed Res. Int..

[155] Leckenby J.I., Furrer C., Haug L., Juon Personeni B., Vögelin E. (2020). A retrospective case series reporting the outcomes of Avance nerve allografts in the treatment of peripheral nerve injuries. Plast. Reconstr. Surg..

[156] Aigner T.B., Haynl C., Salehi S., O’Connor A., Scheibel T. (2020). Nerve guidance conduit design based on self-rolling tubes. Mater. Today Bio.

[157] Bekler H.I., Rosenwasser M.P., Akilina Y., Bulut G. (2010). The use of an absorbable collagen cover (NeuraWrap) improves patency of interpositional vein grafts. Acta Orthop. Traumatol. Turc..

[158] Cooper A., Bhattarai N., Zhang M. (2011). Fabrication and cellular compatibility of aligned chitosan–PCL fibers for nerve tissue regeneration. Carbohydr. Polym..

[159] Guvendiren M., Molde J., Soares R.M.D., Kohn J. (2016). Designing biomaterials for 3D printing. ACS Biomater. Sci. Eng..

[160] Yannas I.V., Burke J.F. (1980). Design of an artificial skin. I. Basic design principles. J. Biomed. Mater. Res..

[161] Zhang X., Li L., Ouyang J., Zhang L., Xue J., Zhang H., Tao W. (2021). Electroactive electrospun nanofibers for tissue engineering. Nano Today.

[162] Wang J., Cheng Y., Chen L., Zhu T., Ye K., Jia C., Mo X. (2019). In vitro and in vivo studies of electroactive reduced graphene oxide-modified nanofiber scaffolds for peripheral nerve regeneration. Acta Biomater..

[163] Niemczyk-Soczynska B., Dulnik J., Jeznach O., Kołbuk D., Sajkiewicz P. (2021). Shortening of electrospun PLLA fibers by ultrasonication. Micron.

[164] Greenfeld I., Zussman E. (2013). Polymer entanglement loss in extensional flow: Evidence from electrospun short nanofibers. J. Polym. Sci. B Polym. Phys..

[165] Zaszczyńska A., Sajkiewicz P.Ł., Gradys A., Tymkiewicz R., Urbanek O., Kołbuk D. (2020). Influence of process-material conditions on the structure and biological properties of electrospun polyvinylidene fluoride fibers. Bull. Pol. Acad. Sci. Tech. Sci..

[166] Fathona I.W., Yabuki A. (2013). One-step fabrication of short electrospun fibers using an electric spark. J. Mater. Process. Technol..

[167] Ravichandran R., Venugopal J.R., Sundarrajan S., Mukherjee S., Sridhar R., Ramakrishna S. (2012). Minimally invasive injectable short nanofibers of poly (glycerol sebacate) for cardiac tissue engineering. Nanotechnology.

[168] Niemczyk-Soczynska B., Gradys A., Sajkiewicz P. (2020). Hydrophilic surface functionalization of electrospun nanofibrous scaffolds in tissue engineering. Polymers.

[169] Taymouri S., Amirkhani S., Mirian M. (2021). Fabrication and characterization of injectable thermosensitive hydrogel containing dipyridamole loaded polycaprolactone nanoparticles for bone tissue engineering. J. Drug Deliv. Sci. Technol..

[170] Thein-Han W.W., Misra R.D.K. (2009). Biomimetic chitosan–nanohydroxyapatite composite scaffolds for bone tissue engineering. Acta Biomater..

[171] Shi Z., Zhong Q., Chen Y., Gao J., Pan X., Lian Q., Cheng H. (2021). Nanohydroxyapatite, Nanosilicate-Reinforced Injectable, and Biomimetic Gelatin-Methacryloyl Hydrogel for Bone Tissue Engineering. Int. J. Nanomed..

[172] Chen Y., Li C., Wang Z., Long J., Wang R., Zhao J., Lai Y. (2021). Self-assembled nanocomposite hydrogels enhanced by nanoparticles phosphonate-magnesium coordination for bone regeneration. Appl. Mater. Today.

[173] Kashte S., Dhumal R., Chaudhary P., Sharma R.K., Dighe V., Kadam S. (2021). Bone regeneration in critical-size calvarial defect using functional biocompatible osteoinductive herbal scaffolds and human umbilical cord Wharton’s Jelly-derived mesenchymal stem cells. Mater. Today Commun..

[174] Mahya S., Ai J., Shojae S., Khonakdar H.A., Darbemamieh G., Shirian S. (2021). Berberine loaded chitosan nanoparticles encapsulated in polysaccharide-based hydrogel for the repair of spinal cord. Int. J. Biol..

[175] Tunesi M., Raimondi I., Russo T., Colombo L., Micotti E., Brandi E., Albani D. (2019). Hydrogel-based delivery of Tat-fused protein Hsp70 protects dopaminergic cells in vitro and in a mouse model of Parkinson’s disease. NPG Asia Mater..

[176] Butcher A.L., Offeddu G.S., Oyen M.L. (2014). Nanofibrous hydrogel composites as mechanically robust tissue engineering scaffolds. Trends Biotechnol..

[177] Ura D.P., Rosell-Llompart J., Zaszczyńska A., Vasilyev G., Gradys A., Szewczyk P.K., Knapczyk-Korczak J., Avrahami R., Šišková A.O., Arinstein A. (2020). The role of electrical polarity in electrospinning and on the mechanical and structural properties of as-spun fibers. Materials.

[178] Kaniuk Ł., Ferraris S., Spriano S., Luxbacher T., Krysiak Z., Berniak K., Angelika Zaszczynska A., Marzec M., Bernasik A., Sajkiewicz P. (2021). Time-dependent effects on physicochemical and surface properties of PHBV fibers and films in relation to their interactions with fibroblasts. Appl. Surf. Sci..

[179] Ghosal K., Augustine R., Zaszczynska A., Barman M., Jain A., Hasan A., Kalarikkalf N., Sajkiewicz P., Thomas S. (2021). Novel drug delivery systems based on triaxial electrospinning based nanofibers. React. Funct. Polym..

[180] Zare P., Pezeshki-Modaress M., Davachi S.M., Zare P., Yazdian F., Simorgh S., Bagher Z. (2021). Alginate sulfate-based hydrogel/nanofiber composite scaffold with controlled Kartogenin delivery for tissue engineering. Carbohydr. Polym..

[181] Mohabatpour F., Karkhaneh A., Sharifi A.M. (2016). A hydrogel/fiber composite scaffold for chondrocyte encapsulation in cartilage tissue regeneration. RSC Adv..

[182] Maharjan B., Park J., Kaliannagounder V.K., Awasthi G.P., Joshi M.K., Park C.H., Kim C.S. (2021). Regenerated cellulose nanofiber reinforced chitosan hydrogel scaffolds for bone tissue engineering. Carbohydr. Polym.

[183] Thieme M., Agarwal S., Wendorff J.H., Greiner A. (2011). Electrospinning and cutting of ultrafine bioerodible poly (lactide-co-ethylene oxide) tri-and multiblock copolymer fibers for inhalation applications. Polym. Adv. Technol..

[184] Hsieh A., Zahir T., Lapitsky Y., Amsden B., Wan W., Shoichet M.S. (2010). Hydrogel/electrospun fiber composites influence neural stem/progenitor cell fate. Soft Matter.

[185] Ghaderinejad P., Najmoddin N., Bagher Z., Saeed M., Karimi S., Simorgh S., Pezeshki-Modaress M. (2021). An injectable anisotropic alginate hydrogel containing oriented fibers for nerve tissue engineering. Chem. Eng. Sci..

[186] Ghouse S., Reznikov N., Boughton O.R., Babu S., Ng K.G., Blunn G., Jeffers J.R. (2019). The design and in vivo testing of a locally stiffness-matched porous scaffold. Appl. Mater. Today.

